# Communication skills training in undergraduate medical education at Charité – Universitätsmedizin Berlin

**DOI:** 10.3205/zma001452

**Published:** 2021-03-15

**Authors:** Rolf Kienle, Julia Freytag, Susanne Lück, Peter Eberz, Sylke Langenbeck, Victoria Sehy, Tanja Hitzblech

**Affiliations:** 1 Charité – Universitätsmedizin Berlin, corporate member of Freie Universität Berlin, Humboldt-Universität zu Berlin, and Berlin Institute of Health, Prodekanat für Studium und Lehre, Team Spezielle Lehrformate, Berlin, Germany; 2 Charité – Universitätsmedizin Berlin, corporate member of Freie Universität Berlin, Humboldt-Universität zu Berlin, and Berlin Institute of Health, Prodekanat für Studium und Lehre, Referat für Studienangelegenheiten, Prüfungsbereich, Berlin, Germany

**Keywords:** communication curriculum, model medicine curriculum, communicative competence, social competence, communication, medical training, faculty development

## Abstract

**Objective: **The objective of this article is a description of the longitudinal communication curriculum in the Model Medicine Curriculum (MSM) at Charité – Universitätsmedizin Berlin. The authors describe the planning and integration of the curriculum into the study program, outline how communicative competence is taught and evaluated in the MSM, and identify which challenges need to be mastered in the process.

**Project description: **Starting with the introduction of the MSM in 2010, students have been spending 102 class hours, spread out over seven semesters, practicing social and communicative competences in the interactive small group format “Communication, Interaction & Teamwork (KIT)”. The course contents are closely linked to the topics covered each semester and increase in complexity over the course of their studies. The contents are selected by the KIT planning group whose members continually check the curriculum’s timeliness and determine any changes. Students as well as instructors have opportunities for evaluating KIT throughout, and their evaluations are taken into consideration as KIT continues to be updated. Instructors from different disciplines teach KIT courses. They participate in mandatory didactic trainings that prepare them to teach KIT. During their 4^th^ and 9^th^ semesters, respectively, students take summative exams that test their communicative competence.

**Results: **According to the semester evaluations by students and instructors, students participating in KIT improved their conversation management skills (students: M=2.2, SD=1.1, instructors: M=1.9, SD=0.7, on a scale of 1-5). In addition, students and graduates rate KIT to be (very) relevant, consider the degree to which it is taught in the MSM to be (very) high, and consider KIT to be a meaningful part of the curriculum.

Students taking the summative exams in their 4^th^ and 9^th^ semesters achieve a mean score of 75.9%, respectively 76.9%, in the purely communicative stations and 82.6%, respectively 83.3%, in the global evaluation of communicative competence in clinical-practical stations.

**Discussion: **Survey and exam results alike indicate that the communication training is well accepted by students and instructors and that the training led to an improvement in general and specific communicative skills.

Due to a lack of control groups or a pre-post design, it has thus far not been possible to unequivocally demonstrate a causal relationship between communicative competence trainings and good test results. Quality control measures, such as trainings for instructors and regular course evaluations, have been designed to address any challenges in the implementation of the communication curriculum at the faculty level.

**Conclusion:** Building on the experience with the Charité’s Reformed Medical Curriculum, a longitudinal, competence-based communication curriculum was integrated into the MSM’s overall curriculum. This measure remedied a gap in the medical training that many graduates of regular study programs had previously bemoaned (Jansen 2010 [1]).

## Zusammenfassung

**Zielsetzung: **Ziel dieses Beitrags ist die Darstellung des longitudinalen Kommunikationscurriculums im Modellstudiengang Medizin (MSM) der Charité Universitätsmedizin Berlin. Dabei werden die Planung und Integration dieses Curriculums in den Studiengang beschrieben, die Lehre und Prüfung der kommunikativen Kompetenzen im MSM und welche Herausforderungen dabei zu meistern sind.

**Projektbeschreibung: **Seit der Einführung des MSM im Jahr 2010 trainieren die Studierenden in 102 Unterrichtsstunden, verteilt über sieben Semester, soziale und kommunikative Kompetenzen in dem interaktiven Kleingruppenunterricht „Kommunikation, Interaktion & Teamarbeit (KIT)“. Die Inhalte dieses Unterrichts sind eng verknüpft mit den Themen des jeweiligen Semesters und nehmen in ihrer Komplexität im Verlauf des Studiums zu. Ausgewählt wurden die Inhalte von der Planungsgruppe KIT, die auch fortwährend die Aktualität des Curriculums prüft und Änderungen beschließt. Für Studierende als auch Lehrende stehen mehrere Möglichkeiten zur Verfügung, KIT zu evaluieren, die ebenfalls in die Überarbeitung miteinfließen. Dozierende aus verschiedenen Fachrichtungen leiten die KIT-Kurse und werden in verpflichtenden didaktischen Schulungen auf diese Aufgabe vorbereitet. Im 4., sowie im 9. Semester erfolgt eine summative Prüfung der kommunikativen Kompetenzen.

**Ergebnisse:** Laut der Semesterevaluationen geben Studierende und Lehrende an, dass die Studierenden in KIT ihre Gesprächsführungskompetenzen verbessern konnten (Studierende: M=2.2, SD=1.1, Lehrende: M=1.9, SD=0.7, Skala 1-5). Außerdem bewerten Studierende bzw. Absolvent*innen KIT als (sehr) relevant, sehen das Ausmaß seiner Vermittlung im MSM als (sehr) hoch an und KIT als sinnvollen Bestandteil des Curriculums.

In den Prüfungen des 4. und 9. Semesters erreichten die Studierenden im Mittel 75.9% bzw. 76.9% in den rein kommunikativen Stationen und 82.6% bzw. 83.3% im Mittel in der globalen Bewertung der kommunikativen Kompetenzen in klinisch-praktischen Stationen.

**Diskussion: **Die Evaluations- und Prüfungsergebnisse weisen darauf hin, dass das Kommunikationstraining von Studierenden und Dozierenden gut angenommen wird und eine Verbesserung allgemeiner und spezifischer kommunikativer Kompetenzen erreicht wurde.

Ein kausaler Zusammenhang zwischen dem Training kommunikativer Kompetenzen und den guten Prüfungsergebnissen konnte durch fehlende Prä-Post-Untersuchungen oder nicht umsetzbare Vergleiche mit Kontrollgruppen bisher nicht eindeutig nachgewiesen werden. Fakultätsinternen Herausforderungen bei der Durchführung des Kommunikationscurriculums, wie z.B. einem sehr großen und heterogenen Pool an Dozierenden, wird mit Qualitätssicherungsmaßnahmen wie Dozierendenschulungen und regelmäßigen Unterrichtsevaluationen begegnet.

**Schlussfolgerung: **Aufbauend auf den Erfahrungen im Reformstudiengang der Charité wurde mit Einführung des Modellstudiengangs ein längsschnittliches, kompetenzbasiertes Kommunikationscurriculum in das Gesamtcurriculum integriert. Dadurch konnte ein Ausbildungsdefizit, das von vielen Absolvent*innen von Regelstudiengängen beklagt wurde (Jansen 2010 [1]), behoben werden.

## 1. Introduction

For medical staff, communicative and social competencies represent a basic qualification that should already be developed during their study course [2]. 

At the Charité – Universitätsmedizin Berlin, a longterm communication curriculum was integrated as part of the Reformed Medical Curriculum (RSM) of 1999. Building on the work of the Reformed Medical Curriculum Working Group and using the so-called model clause (§ 41) of the German licensing regulations for physicians as a foundation, the RSM was implemented in 1999 [3] and existed alongside the Traditional Curriculum until 2010. In the RSM, students’ communicative and social competence were being cultivated through the frequent hands-on use of the small group format “interaction”, which includes conversations with simulated patients, respectively simulated participants (SPs) [4]. Students acquired and strengthened general and specific communicative and social skills by participating in progressive learning exercises as part of the longitudinal curriculum [5]. The respective course content was closely interwoven with other classes and was covered on the summative exams. Exam results revealed the effectiveness of the “interaction” format as it led to a growth in communicative competence even if that growth did not develop evenly across all semesters [6].

When funding for the RSM was about to expire, a decision was made to blend the RSM with the regular course of study. Starting with the 2010/2011 winter semester, Charité would offer the Model Medicine Curriculum (MSM) as its only study program in medicine. The newly formed study program showed a continuity of certain principles and elements of the Reformed Medical Curriculum. They included the curriculum structure in the form of interdisciplinary modules, a strong focus on competence, practice-oriented exam formats (Objective Structured Clinical Examination (OSCE)), and patient contact from an early phase of the study program.

However, the differences between RSM and MSM, including content changes in the modules, fewer class hours dedicated to practicing communicative and social competences, and a tenfold increase in students rendered adjustments and a new design of the “interaction” format unavoidable. The new class format was named “Communication, Interaction, Teamwork” (KIT).

This article focuses on the ways in which more than 600 newly enrolled students each year are taught communicative and social skills as part of a longitudinal strategy, how they acquire these skills, and how their skills are being evaluated. The article outlines how students and instructors evaluate the curriculum and how the students perform on their communicative competence exams. The article also focuses on the challenges in the implementation and quality control of the curriculum and on the measures that Charité has developed to meet those challenges.

## 2. Project description

### 2.1. Framework and structure 

The KIT course consists of 102 class hours distributed over 32 class meetings. Classes are scheduled during seven of the ten semesters of the medical study program, and meet every two or four weeks, all in all four or six times per semester.

This course schedule represents the kind of longitudinal communication curriculum recommended by research [7] while allowing close interdisciplinary connections to the content of other courses (see attachment 1 ). The study program consists of 40 interdisciplinary modules that teach knowledge and clinical-practical skills in lectures, seminars, and practical training sessions. Students subsequently practice communicative competence relevant to these contents in KIT. Students apply and build on the clinical-practical and communication skills they have developed in course settings like physical examination courses, clinical electives, and internship blocks. Having participated in those courses, students then reflect on their experiences with KIT to foster the development of their professional identities. 

#### 2.2. Didactics

Classes are held in small groups of nine students and one instructor. Since the same instructor teaches all units during any given semester, the instructor can guide group processes and strengthen cohesion among the group members. Instructors hold graduate degrees in medicine, psychology, social sciences or the humanities, and work in various Charité departments.

The small group size allows instructors to teach in an interactive manner. At the same time, learning objectives and course contents are highly standardized, and instructors follow manuals to teach. The manuals contain an overview of learning objectives, a short description of the respective communication model to be discussed, a course schedule, comprehensive guidelines for group discussions, role play and group activities, as well as suggestions for relevant online materials and films.

The majority of class time is dedicated to practical exercises [8]. Simulated consultations with SP and structured feedback take place in 21 of 32 class meetings. The pool of SPs contains approximately 140 persons between the ages of 18 and 80. Prior to their performances, SPs are taught how to present their characters and how to offer feedback. The goal of this strong hands-on training is to teach students how to recognize elements of good conversation management and how to practice these elements themselves. For example, students analyze videos of doctor-patient interactions and practice these skills in hands-on exercises (role plays with or without SPs) [2].

#### 2.3. Human resources

A research associate in the Office of the Vice Dean of Research for Teaching and Learning was tasked with developing the KIT curriculum and class schedule. The curriculum is not guided by the interests of individual disciplines or subject areas. Rather, the research associate founded an interdisciplinary group (“KIT Planning Group”) that serves as a kind of scientific advisory board and includes representatives from medical psychology, medical sociology, education, general medicine, the simulated patient program, and the student body. 

#### 2.4. Learning 0bjectives and contents

The process of developing and implementing the curriculum occurs based on an approach by Kern [9]: The learning objectives closely follow the Basel consensus statement and were developed by the KIT planning group [10]. The national competence-based catalogue of learning objectives (NKLM) [http://www.nklm.de] did not exist when KIT was first developed. However, a comparison with chapter 14c (Managing Doctor Patient Conversations) of the NKLM shows that the competence skills (level 1) agree 100%, and that the individual competence skills (level 2) agree 72%. All learning objectives refer to the theoretical models on which they are based (see attachment 1 ). The contents and teaching methods are organized in an increasingly complex and challenging fashion regarding the patient’s personality, medical condition, and the required communication skills. 

#### 2.5. Exam formats

Students’ communicative competence is evaluated via OSCE after the 4^th^ and the 9^th^ semesters, respectively. The OSCE stations test clinical-practical skills as well as communicative competence. Communication skills make up 30% of the overall evaluation and are measured using the Berlin Global Rating Scale (BGR) [11]. Each of the two OSCE contains one station that exclusively assesses communication skills. Literature-based checklists [12] are used to evaluate these stations. Exam results are issued in the form of grades between 0 and 100%, with 60% as the threshold for a passing grade. The SPs do not provide any feedback on the students’ exams.

#### 2.6. Quality control 

Several measures are taken to ensure quality control and quality development. They are described below.

##### 2.6.1. Instructor trainings

Two mandatory trainings for instructors represent an important parameter for ensuring quality control: the foundational training “Working with simulated participants” and a semester-specific, in-depth training. The foundational training [13] comprises ten course units and contains information about the KIT curriculum, the use of SPs, and group dynamics, as well as methods for small group work. Several mock classroom sessions with subsequent reflection form a core part of the training. The specific trainings of five course units each correspond to the respective topics covered during a specific semester. Using the manuals, participants discuss course units, SP cases and methods, and enact some of them. Participants can only obtain the qualification of “qualified instructor” and become KIT instructors once they have successfully completed the two trainings. Instructors have opportunities to participate in additional, optional trainings, such as, shadowing experienced instructors, receiving didactic feedback, or participating in topic-specific trainings.

Training evaluations show that participants are consistently very pleased with the trainings: From 2012 to 2017, 368 participants evaluated the foundational training and subsequently agreed that they were able to facilitate KIT sessions (M=1.7, SD=0.6) and to prepare and evaluate SP conversations (M=1.4, SD=0.6; on a scale where 1=completely agree and 5=do not agree). Multiple reports based on the shadowing of 60 instructors indicate that at least 75% of the instructors met two thirds of the quality standards for KIT instruction [14]. 

##### 2.6.2. Contents and SP cases 

The KIT Planning Group decides on any necessary curriculum changes owed to societal developments and medical progress. For example, the topics “Interprofessional Collaboration” and “Digitalization in Medicine” were added to the curriculum.

##### 2.6.3. Possibilities for evaluating learning 

Different kinds of feedback by instructors and students serve to continually optimize the curriculum, SP cases, and course materials:

At the end of each semester, students and instructors evaluate the KIT teaching format. Starting with the winter semester 2015/2016, a global item was added that asks to what extent the students’ conversation management skills had improved thanks to KIT. Additionally, during the winter semester 2017/2018, all students were asked to participate in a survey that measured their satisfaction with the study program. A survey for graduates was offered during the summer 2018 semester. In both surveys, participants were asked to rate the relevance of communication skills and the extent to which these skills were taught as part of the study program in medicine at Charité. Furthermore, in the first quarter of 2020 the group representing the student body (FSI) conducted a survey of all medical students at Charité. The survey contained an item that asked students to state to what extent KIT, alongside other learning formats, represents a meaningful part of the MSM curriculum.

In addition to verbal feedback or feedback via e-mail, students can share anonymous feedback on courses via Charité’s Teaching Incident Reporting System (TIRS).

Members of Charité’s FSI and the student representatives of the modules serve as contact persons for student matters and share those in KIT. For example, based on students’ requests, additional training materials for Motivational Interviewing (MI) were created [15] and were made available to the students.

## 3. Results

The results of the evaluations show how students and instructors evaluate the contents of KIT as part of their studies and whether the extent to which they are taught corresponds to the meaning that the students assign to them. Students’ actual performances can be measured via their objectively graded exams. 

### 3.1. Evaluation and survey results 

The results of the student and instructor surveys outline in 2.6.3 are described below.

#### 3.1.1. Semester evaluations

Student and instructor evaluations conducted between the winter semester 2015/16 and the summer semester 2019 were evaluated for this study. Return rates ranged from 4.7% to 47.1% among the students (with a mean value of 34.7%) and from 14.7% to 85.7% among the instructors (with a mean value of 54.9%). For an item asking participants whether KIT led to improved conversation management skills (using a five-point-scale where 1=completely agree and 5=completely disagree), student responses showed a mean value of 2.2 (SD=1.1) and instructor responses showed a mean value of 1.9 (SD=0.7). 

##### 3.1.2. Survey of graduates and study program satisfaction survey

**Figure 1:**
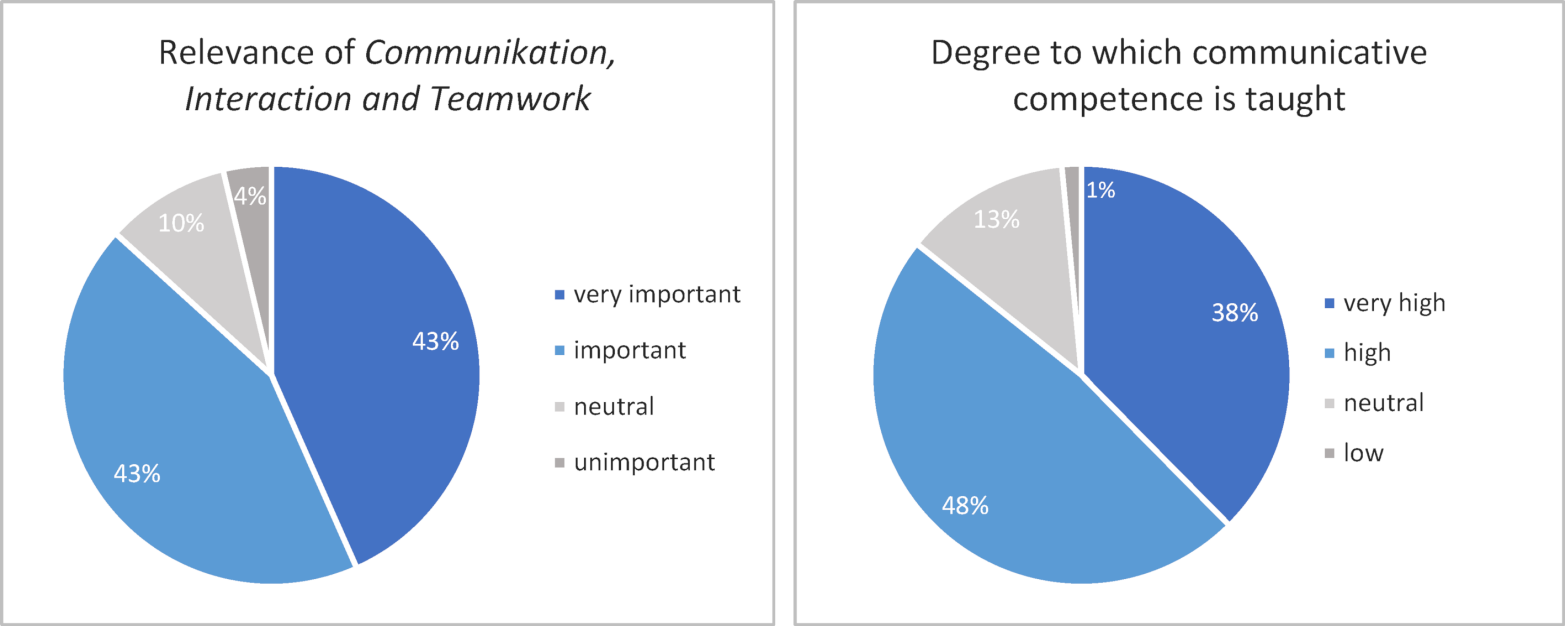
Percentaged distribution of responses from the graduate survey (2018).

**Figure 2:**
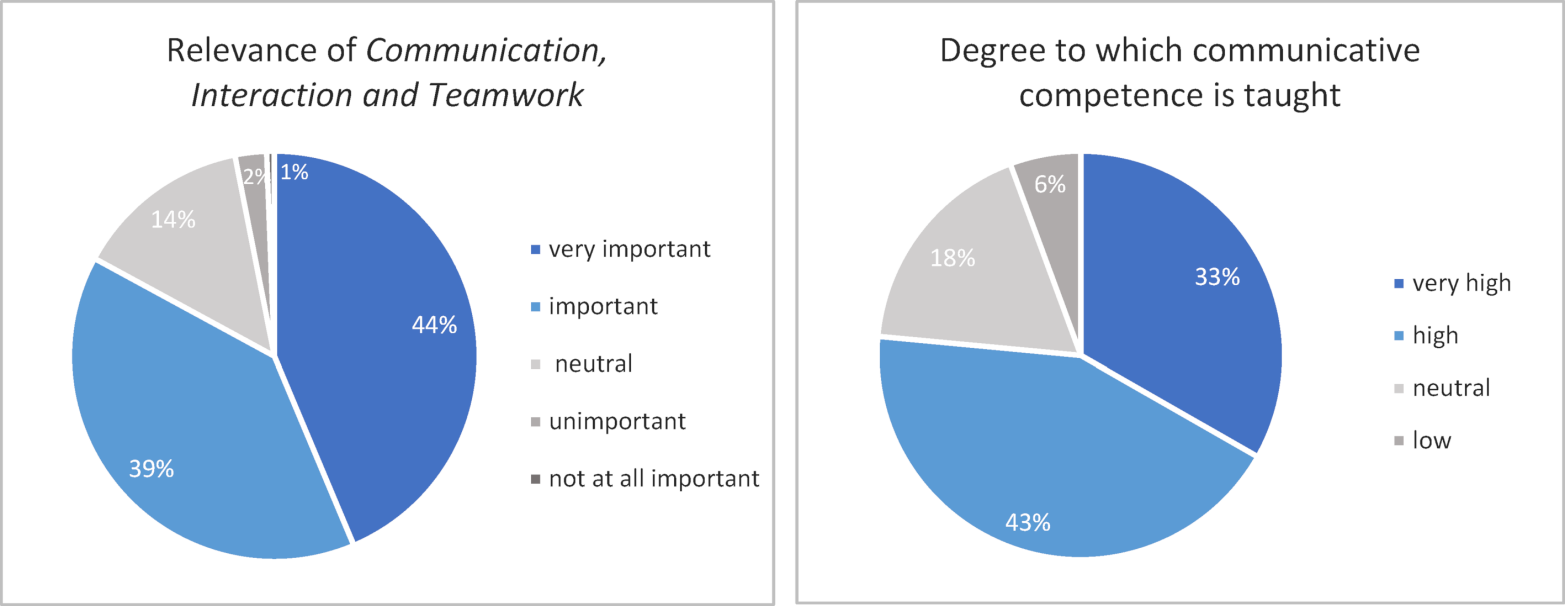
Percentaged distribution of responses from the student satisfaction survey (winter semester 2017/18).

33% of the graduates (n=139) participated in the survey conducted in 2018, 35% of the students (n=1047) participated in the study satisfaction survey administered during the 2017/18 winter semester. Students from all semesters participated equally in the survey (from 6% to 13% per respective semester), with the exception of students in semesters 12 and 13 (0.8% and 0.1%, respectively). The survey results are depicted in figure 1 (Fig. 1) and figure 2 (Fig. 2), below. They indicate that students consider the contents to be very important, and that they perceive communicative competence to play a large role in their study program. 

##### 3.1.3. FSI survey

**Figure 3:**
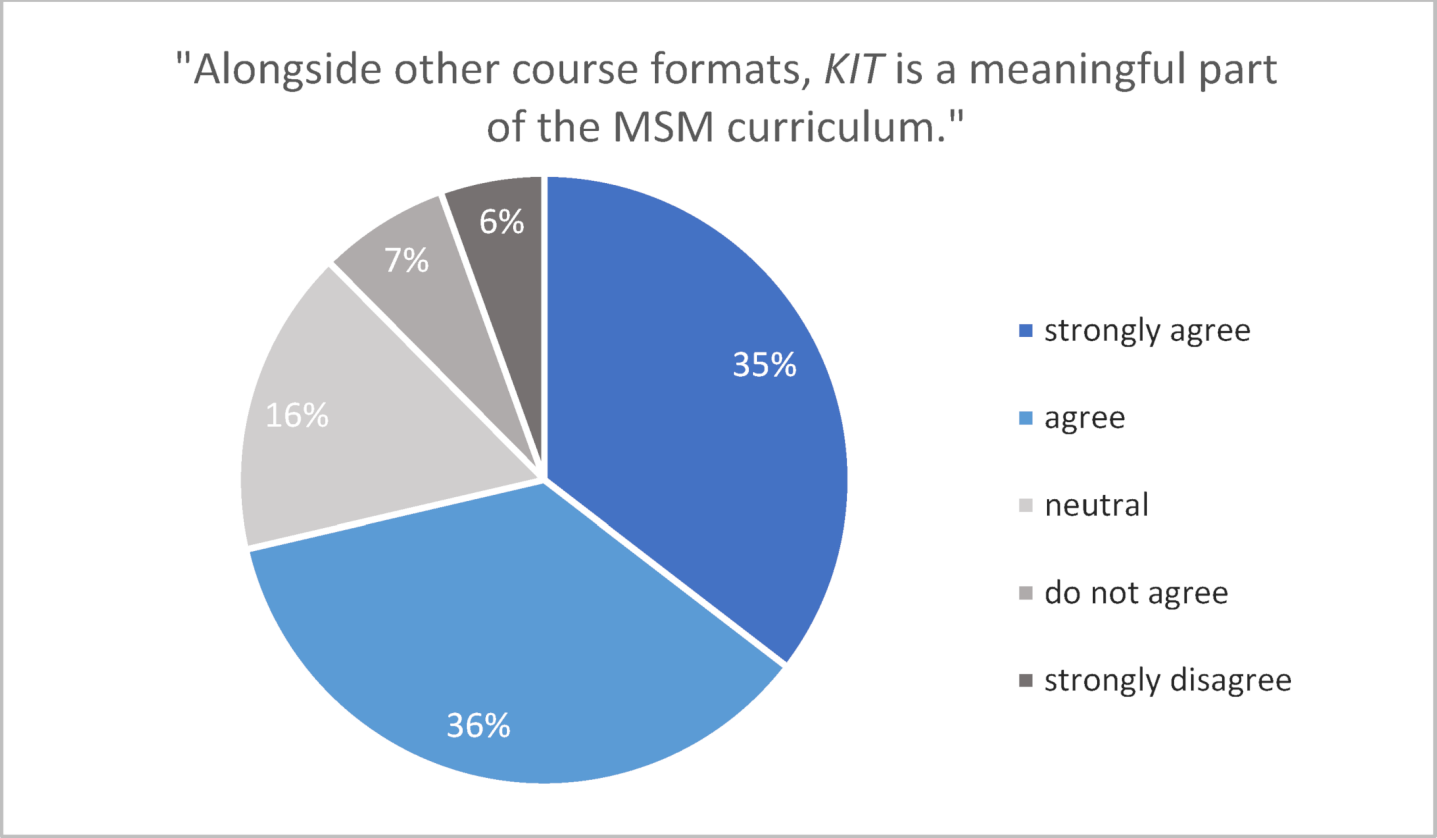
Percentaged distribution of responses from the FSI survey (winter semester 2019/20).

The survey was administered to students in semesters 2-10 and in their clinical internship year (PJ). 824 individuals participated (return rate: 19.2%). The majority of survey takers stated that KIT is a meaningful part of their study program (see figure 3 (Fig. 3)).

#### 3.2. Exam results

**Figure 4:**
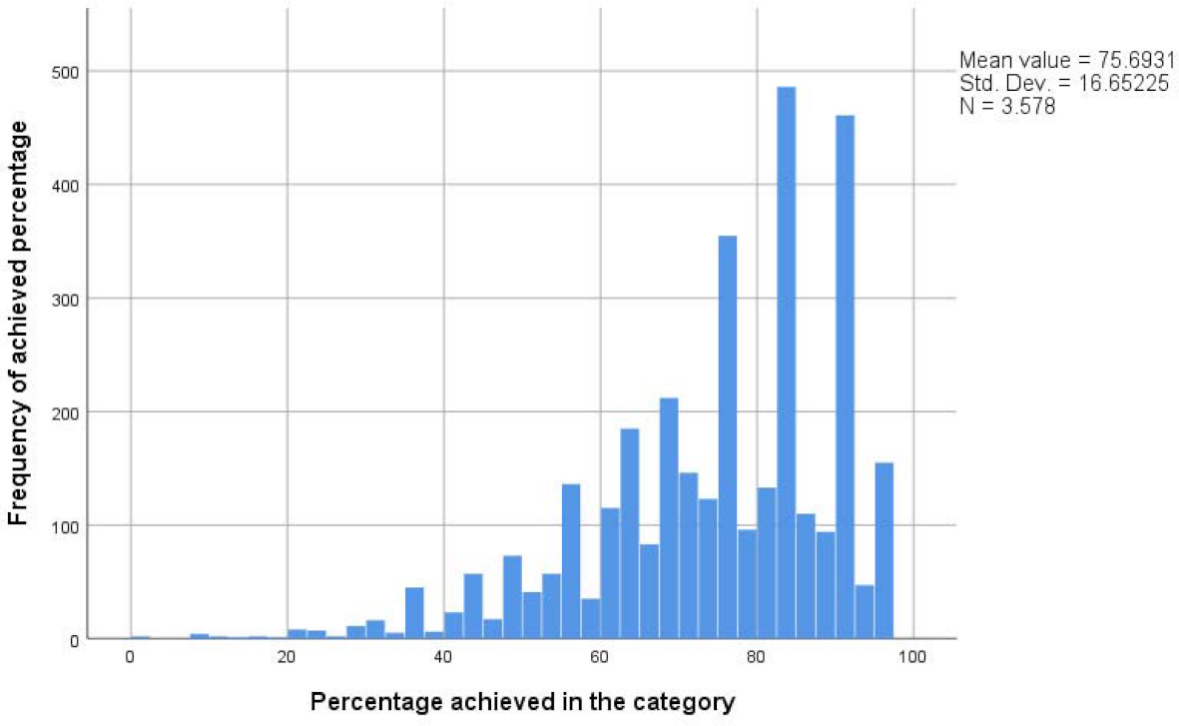
Frequency distribution of the percentage achieved in the MI category (4^th^ semester OSCE) since summer semester 2013.

**Figure 5:**
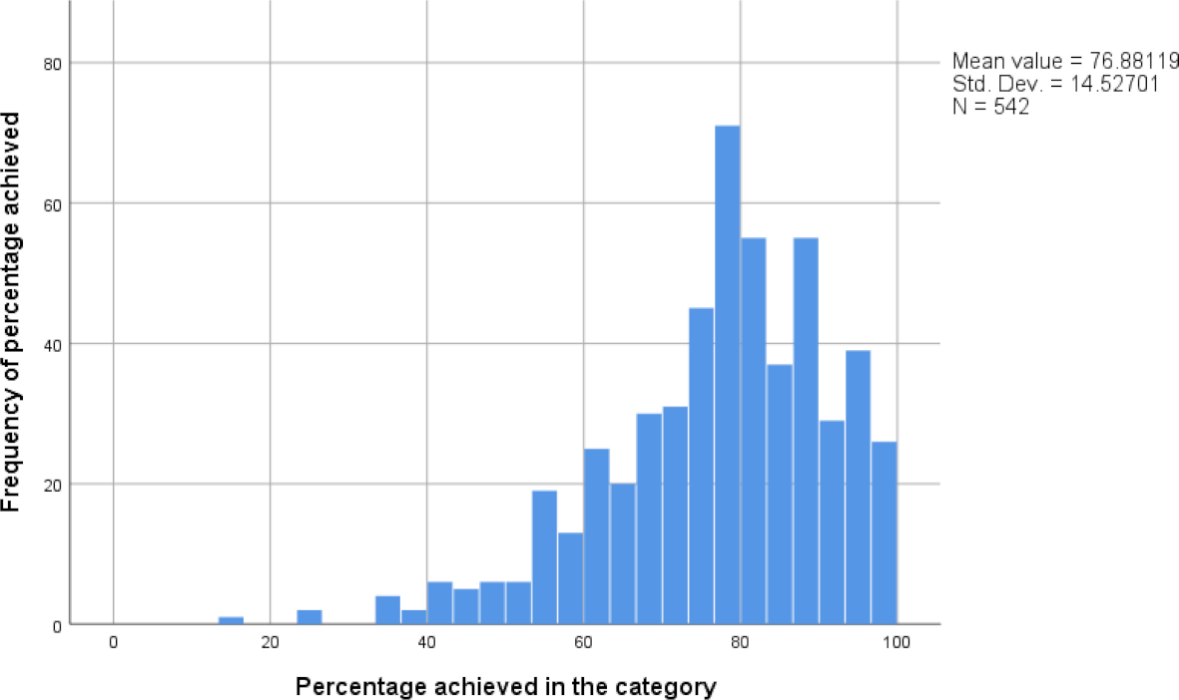
Frequency distribution of the percentage achieved in the category of delivering bad news (9^th^ semester OSCE) since the summer semester 2019.

During the summer 2013 semester, motivational interviewing was introduced as a KIT exam station in the 4th semester OSCE. Ever since then, students have achieved a mean score of 75.7% (SD=16.7, n=3578, see figure 4 (Fig. 4)). This tendency corresponds to the achievements in other stations that foreground clinical skills (78.4%, SD=6.7). Ever since the 2019 summer semester, delivering bad news using the SPIKES model has been examined as a KIT exam station in the 9th semester OSCE. Students achieved a mean score of 76.9% in that station (SD=14.5, n=542, see figure 5 (Fig. 5)). The mean score of all other exam stations employed in the 9th semester OSCE is 79.7% (SD=7.7).

The BGR is used to determine the overall communicative competence. Since 2013, the mean percentage achieved for the 4th semester OSCE has been 82.6% (SD=7.6). Since the winter semester 2014/15, when it was first implemented in the 9th semester OSCE, the mean percentage achieved has been 83.3% (SD=6.3).

## 4. Discussion

### 4.1. Evaluation

The overwhelming majority of graduates who completed the study program satisfaction survey considers communicative competence an important if not very important aspect of medical practice. Other studies in German-speaking countries [16] also reveal a rather positive attitude among students toward communication skills and suggest that communication skills trainings can strengthen students’ positive attitudes as well as reduce negative attitudes toward acquiring communicative competence [17]. 

While graduates of regular study programs have judged the degree to which communication skills are taught as too low [1], MSM students judged the degree to be very high, respectively, high. These findings suggest that in comparison to the regular study program, the MSM meets the students’ needs for comprehensive training in communication skills.

In surveys that are conducted each semester, students and instructors report that the students’ conversation management skills have improved due to KIT. Hence, the training results in actually perceived learning effects.

On a cautionary note, one needs to acknowledge that the items in all the surveys are phrased in a rather general manner, and that one cannot use them to infer any information about specific aspects of KIT. Furthermore, since some of the return rates are rather low one cannot exclude the possibility that respondents tended to have a more positive bias toward the program whereas those with more critical perspectives did not participate in the survey.

#### 4.2. Exams

The students’ exam results indicate that the students possess good general as well as specific communication skills. An evaluation of the BGR, however, shows that rather than using the full spectrum of the grading scale, examiners tend to only use the upper spectrum of the scale. This can suggest a tendency to grade rather leniently. The exam results that test specific communication skills shows that most students are well able to conduct a conversation applying MI or SPIKES principles. In order to document that test results are a clear indication of KIT’s success one would have to test students before and after their participation in KIT or offer tests to a control group of students. Neither measure has been implemented thus far. 

#### 4.3. Integrating the communication curriculum

Partly due to the fact that KIT is offered in seven out of ten semesters of study, it was possible to comprehensively integrate KIT’s contents into the overall curriculum. This is rare compared to other medical school curricula in German-speaking countries that usually focus on instruction during the second or third year of study [18]. KIT’s integration into the overall curriculum comes with a cost: For example, in order to create connections to other courses, 4^th^ semester students are already taught MI although MI is a rather challenging concept to comprehend at this early stage of one’s studies. Similarly, there are some limitations in always keeping the curriculum up to date. While the topic “Digitalization in Medicine” could be integrated into the curriculum, only a small portion of the medical students have opportunities to study the topic “Interprofessional Collaboration” along with students of different professional study programs. Due to legal and organizational reasons, this topic is mostly taught in mono-professional groups.

#### 4.4. Quality control

Program managers strive for a high degree in standardization regarding course content and course quality. However, instructors work in different departments at Charité and, depending on their respective field, differ in their previous experience with small group work and communication skills trainings. Moreover, there exists a high level of fluctuation among instructors. Instructor manuals have been produced to address these issues, and 100% of instructors self-reported in their evaluations that they used the manuals when preparing for class. In addition, all instructors are obliged to participate in mandatory trainings. However, the following question remains unanswered: Why do student evaluations of instructors’ teaching skills differ so widely? It is possible that while trainings provide the didactic skills needed for teaching, but that the trainings alone do not compensate for differences in prior knowledge or teaching skills.

Thanks to surveys as well as anonymous feedback systems one can identify the needs of students and instructors as the so-called “end consumers”. These measures allow one to quickly respond to problems and to continually improve the curriculum.

#### 4.5. Faculty development

To date, no systematic studies exist that reveal to what extent the students’ participation in the communication training at Charité has led to better communication in healthcare. However, student evaluations and individual case reports suggest that it has done so.

Developing a longitudinal communication curriculum consisting of 102 learning units has de facto increased the significance of developing this competence. However, we do not have any results yet that show to what extent faculty recognize the relevance of this training. Individual case reports of instructors who have completed the MSM suggest that they consider the communication training to be an indispensable part of the study program. 

## 5. Conclusions

As part of introducing the MSM at Charité, the longitudinal and competence-based communications curriculum, KIT, was integrated into the overall curriculum. To this end, having central office for coordination and further development, as well as a panel of experts without any monetary or discipline-specific dependencies proved to be essential. Compulsory trainings for instructors as well as standardized course manuals and course materials significantly contribute to quality control. Students’ and instructors’ evaluations of KIT are positive, and exams testing communicative competences document positive results throughout.

The development of the communication curriculum at Charité and the implemented quality measures aim to show students and instructors that acquiring communicative and social competence is not merely an optional additional qualification, but rather, a necessary basic qualification for working in the medical profession [19].

## Authors

Rolf Kienle and Julia Freytag share the first authorship. 

## Acknowledgements

We thank our colleagues in the Quality Management Team, our colleagues at the Dieter Scheffner Fachzentrum, and the students at FSI Berlin for sharing their survey data with us. In addition, we thank the students and instructors who participated in the surveys, thereby making an important contribution to quality management. 

## Profiles

**Name of the location: **Charité – Universitätsmedizin Berlin, corporate member of Freie Universität Berlin, Humboldt-Universität zu Berlin, and Berlin Institute of Health

**Study program/occupation: **Human Medicine

**Number of students per year and/or semester: **320 students are enrolled each winter and each summer semester.

**Has a longitudinal curriculum covering communication been implemented?** Yes

**At which semester levels are communicative and social competencies taught?** In semesters 1, 2, 4, 5, 6, 8 and 9

**Which teaching formats are used?** Interactive small group training of 9 students per lecturer (including working with SP)

**In which semesters are communicative and social competencies assessed (formative or pass/fail or graded)?** Summative, graded exams in semester 4 and 9 using the Berlin Global Rating Scale and checklists

**Which assessment formats are used?** Objective Structured Clinical Examination (OSCE)

**Who (e.g., hospitals, institution) is in charge of development and implementation?** Development/Quality Assurance: Vice Dean's Office for Teaching and Learning, “KIT team and Simulated Patient Program” (supported by an interdisciplinary group of experts (“KIT planning group”)

**Implementation: **Lecturers from different clinics and institutes 

## Current professional roles of the authors

- Rolf Kienle, licensed psychotherapist, works as a research associate in the Vice Dean's Office for Teaching and Learning and oversees the design, development and quality assurance of the small group teaching format “Communication, Interaction, Teamwork” (KIT). He is a member of the “KIT planning group”.
- Julia Freytag is a psychologist (M.Sc.) and as a research associate in the Simulated Patient Program responsible for SP trainings, role development, quality assurance and the training of KIT lecturers. She is a member of the “KIT planning group” and, in this function, also in charge of KIT quality assurance.
- Susanne Lück, B.Sc. Psychology, is responsible for coordination and quality assurance in the Simulated Patient Program, for SP trainings and role development.
- Peter Eberz, MA/PGDip – Landscape Architecture, actor and circus teacher, is responsible for coordination and quality assurance of the Simulated Patient Program, SP trainings and role developments and trains KIT lecturers.
- Sylke Langenbeck is a psychologist (Dipl.-Psych.) and works as a research associate at the Office of Student Affairs, Team Assessment, at Charité-Universitätsmedizin Berlin. She is responsible for the coordination, quality assurance, data evaluation and analysis of OSCE as well as the development of OSCE stations. She also conducts SP trainings in collaboration with the Simulated Patient Program.
- Victoria Sehy is a psychologist (M.Sc) and works as a research associate at the Office of Student Affairs, Team Assessment, at Charité-Universitätsmedizin Berlin. She is responsible for the coordination, quality assurance, data evaluation and analysis of OSCE as well as the development of OSCE stations. She also conducts SP trainings in collaboration with the Simulated Patient Program.
- Tanja Hitzblech, certified pedagogue, leads the team responsible for organizing the small group teaching formats “Problem-Based Learning” and “Communication, Interaction, Teamwork” (KIT) and the Simulated Patient Program. Prior to that, she worked in the project management of the Model Medicine Curriculum (MSM) as curriculum and faculty developer (incl. change management).

## Competing interests

The authors declare that they have no competing interests. 

## Supplementary Material

Overview over the content and the theoretical foundations of KIT and its integration into the Model Medicine Curriculum at Charité
