# Supplementary material for: Communication skills training in undergraduate medical education at Charité – Universitätsmedizin Berlin
Source: GMS J Med Educ. 2021 Mar 15;38(3):Doc56. doi: 10.3205/zma001452 (PMC7994867; doi:10.3205/zma001452)
Supplement: Overview over the content and the theoretical foundations of KIT and its integration into the Model Medicine Curriculum at Charité [file JME-38-3-56-s-001.pdf]

Attachment 1: *Overview over the content and the theoretical foundations of KIT and its integration into the Model Medicine Curriculum at Charité*

Semester	Class meetings/ Class hours (each 45 minutes)	Content of the communication skills training	Framework and theoretical foundations	Integration into the curriculum (links to other lectures and courses of the curriculum)
1	1 meeting / 3 class hours (in total)	Group interaction	Feedback rules (Mariana G. Hewson), Rangdynamic Position Model (Raoul Schindler)	Working as a team in problem-based learning
	3 meetings / 9 class hours (in total)	Conversation management skills, history taking, nonverbal communication	Communication Square (Friedemann Schulz von Thun), patient-centered communication (Carl Rogers), "WWSZ" (waiting, repeating, reflecting, summarizing), methods of history taking	Basic course on physical examination, Excercises on social perception (facial expression, hand gestures, behavior)
2	4 meetings / 12 class hours (in total)	History taking, doctor-patient-relationship	Social roles of doctor and patient (Talcott Parsons); paternalistic, shared, and informative model; stereotypes; gender; intercultural communication	Basic courses on physical examination; seminars and practice sessions about topics of medical psychology and medical sociology (intercultural competencies, doctor-patient-relationship)
4	4 meetings / 12 class hours (in total)	Motivational Interviewing, health-related behavior, changing lifestyle, adherence	Motivational Interviewing (Miller & Rollnick), transtheoretical model (Prochaska & DiClemente)	Advanced courses on physical examination; lectures and practical training about stress models und stress management
5	4 meetings / 12 class hours (in total)	Interprofessional collaboration, team communication, communication during ward rounds, history taking in psychosomatic medicine	stages of group development (Bruce Tuckman), model of conflict escalation (Friedrich Glasl), biopsychosocial model, psychodynamic explanations (transference and countertransference)	Bedside teaching in the module „Psyche and pain“; lectures and seminars about the biopsychosocial model and different methods of psychotherapy
6	4 meetings / 12 class hours (in total)	Error management and patient safety, sexual history taking	Critical Incident Reporting System (CIRS), London-Protocol, taboo as a sociological term	Bedside teaching in emergency medicine and with patients from gynecology and urology
8	6 meetings / 18 class hours (in total)	Consultation and informed consent, history taking in neurology and psychiatry, multiperson interview	Legal frameworks for obtaining informed consent to treatment, psychopathologicial asessment, diagnostic of suicidal tendencies and suicide prevention	Bedside teaching with patients from neurology and psychiatry; lectures and seminars about psychiatry and neurology

9	6 meetings /<br>24 class hours (in total)	Family sessions in paediatrics,<br>emotionally challenging situations,<br>domestic violence,<br>breaking bad news	NURSE-model,<br>CALM-model,<br>Power and Control Wheel,<br>SPIKES-protocol (Baile & Buckman)	Bedside teaching with patients from pediatrics and geriatrics; lectures and seminars about history taking in pediatrics, domestic violence, geriatrics

Explanatory notes to table:

The integration of KIT into the curriculum shall be outlined in an exemplary manner by depicting how the topic of medical care for dementia patients is taught. The students taking the module “psychiatry” in the 8th semester first attend lectures on diagnostic and treatment, symptoms, and epidemiological aspects. Additionally, students attend a lecture on neurological tests and are provided with online learning materials to deepen their knowledge. In the following KIT class meeting the students practice two competences: Firstly, they are trained to consider the specific communication needs of a person who is cognitively impaired by dementia. Secondly, they are trained to conduct a patient-centred and goal-oriented interview with a patient suffering from dementia and their daughter. This is realized by an interview with two simulated participants (mother/father [patient] and daughter) in the primary care setting. Subsequently the students apply the acquired the medical knowledge and the communication competencies in bedside teaching in psychiatry.
